# Compassion training in healthcare: what are patients’ perspectives on training healthcare providers?

**DOI:** 10.1186/s12909-016-0695-0

**Published:** 2016-07-11

**Authors:** Shane Sinclair, Mia-Bernadine Torres, Shelley Raffin-Bouchal, Thomas F. Hack, Susan McClement, Neil A. Hagen, Harvey M. Chochinov

**Affiliations:** Faculty of Nursing, University of Calgary, 2500 University Dr. NW, Calgary, AB T2N 1N4 Canada; College of Nursing, Faculty of Health Sciences, University of Manitoba, 66 Chancellors Cir, Winnipeg, MB R3T 2N2 Canada; Department of Oncology, Cumming School of Medicine, Health Sciences Centre, Foothills Campus, University of Calgary, 3330 Hospital Drive NW, Calgary, AB T2N 4N1 Canada; Department of Psychiatry, University of Manitoba, PsycHealth Centre PZ433-771 Bannatyne Avenue, Winnipeg, MB R3E 3N4 Canada

## Abstract

**Background:**

The purpose of this qualitative study was to investigate advanced cancer patients’ perspectives on the importance, feasibility, teaching methods, and issues associated with training healthcare providers in compassionate care.

**Methods:**

This study utilized grounded theory, a qualitative research method, to develop an empirical understanding of compassion education rooted in direct patient reports. Audio-recorded semi-structured interviews were conducted to obtain an in-depth understanding of compassion training from the perspectives of hospitalized advanced cancer patients (*n* = 53). Data were analyzed in accordance with grounded theory to determine the key elements of the underlying theory.

**Results:**

Three overarching categories and associated themes emerged from the data: compassion aptitude, cultivating compassion, and training methods. Participants spoke of compassion as an innate quality embedded in the character of learners prior to their healthcare training, which could be nurtured through experiential learning and reflective practices. Patients felt that the innate qualities that learners possessed at baseline were further fashioned by personal and practice experiences, and vocational motivators. Participants also provided recommendations for compassion training, including developing an interpersonal relationship with patients, seeing the patient as a person, and developing a human connection. Teaching methods that patients suggested in compassion training included patient-centered communication, self-reflection exercises, and compassionate role modeling.

**Conclusions:**

This study provides insight on compassion training for both current and future healthcare providers, from the perspectives of the end recipients of healthcare provider training – patients. Developing a theoretical base for patient centred, evidence-informed, compassion training is a crucial initial step toward the further development of this core healthcare competency.

## Background

**Fig. 1:**
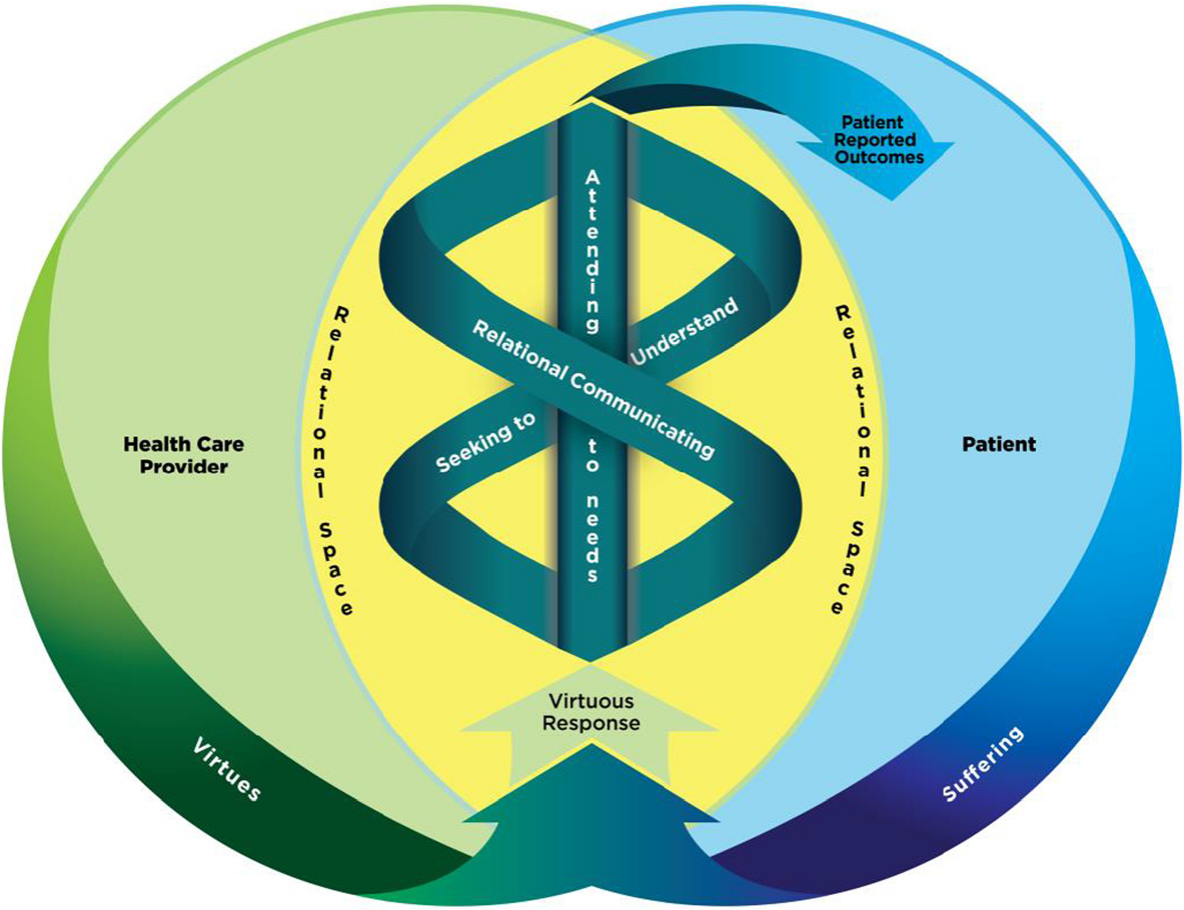
Compassion Model: Compassion in Clinical Practice [17]

While the importance of compassion has been extolled in fields such as psychology, social work, and theology, it is now being recognized for its positive impact in healthcare, most notably in advanced illness [1–11]. As its significance becomes increasingly recognized in enhancing quality patient care, wellbeing and overall quality of life, compassion and compassionate care are emerging as a competency that healthcare providers are expected to deliver [5, 7, 12–19]. Unfortunately, this call to action has been accompanied with little guidance on the feasibility, skills and methods of compassion training, to aid healthcare educators, students and healthcare providers in addressing this educational and practice issue. Can compassion be taught? What are the best methods to train future healthcare workers in compassionate care? What are the requisite attitudes, knowledge and skills in competent compassionate care? The current study is a secondary analysis of a data subset from a broader grounded theory study investigating patients’ understandings and experiences of compassion [17], which produced a clinical model of compassion (Fig. 1) and an empirical definition of compassion-- *a virtuous response that seeks to address the suffering of a person through relational understanding and action*. The research question that guided this secondary analysis was, ‘What are patients’ perspectives on training healthcare providers in compassion?’.

Although compassion is increasingly espoused as a core competency of healthcare education and healthcare delivery, the learner attributes and competencies of a compassionate care provider are poorly understood [3, 19, 20]. In addition to this knowledge gap, a significant practice gap in compassionate care has emerged, marked by critical incidences where compassion was lacking, leading to a system-wide healthcare reform calling for the reintegration of compassion into healthcare delivery and education [56, 18, 19]. This call to action has been particularly pronounced in the United Kingdom, where compassion was recently identified as an outcome of high-quality healthcare education [6], requiring healthcare educators to “clearly evidence how users [patients] and carers contribute to programme delivery and design” ([6] p.34).

Despite recent recommendations to include the patient voice in the development of compassionate care education, a scoping review of compassion in the healthcare literature [21] yielded no studies that directly reported on patients’ perspectives on the importance, feasibility, and teaching methods associated with compassionate care. One study investigated patients’ views of compassionate nursing care, but did not directly inquire as to whether and how these practices could be taught [22]. A number of articles reported on healthcare provider, educator and student perspectives on the feasibility and teaching methods associated with compassion training. These studies, along with a number of theoretical articles, concluded that while teaching compassion is feasible, it seems reliant on the innate qualities that learners possess prior to their healthcare education [20, 23–26]. These papers identified a variety of teaching methods that may be effective in cultivating compassion, including clinical simulation [27, 28], reflective essays [25, 29], role modeling [30], direct interaction with dying patients [22, 27], and reflective practice techniques [31–33].

Recent studies in the neurosciences have differentiated functional brain plasticity in participants who received contemplative training intended to generate compassionate feelings to others (extending caring feelings) versus participants who were trained to have empathetic feelings to others (resonating with others suffering) [34–36]. The authors reported that in contrast to empathetic meditators that activated regions of the brain associated with negative affect and aversion, compassion focused meditation activated regions associated with reward, love and affiliation, suggesting that a distinguishing feature of compassion training is its buffering effect against burnout. While providing insight into the importance of cultivating compassionate feelings to others, these studies provide limited insight related to the clinical skills, behaviours and teaching methods associated with ‘applied’ compassion, which is important as action, has been identified as a defining feature of compassion [17].

Despite current and future healthcare providers’ desire to provide compassionate care and the requirement for healthcare education providers to incorporate compassion into the curriculum—educators, students and healthcare providers are provided little guidance on how to develop competencies in compassionate care. These challenges underscore the importance of developing evidence-informed compassion training programs that are clinically informed and relevant to the individuals they are targeted toward—patients themselves.

## Methods

### Study population

This study is a subset of a larger study whose methods have been reported in detail elsewhere [17]. In conducting a scoping review of compassion in healthcare [21] to inform protocol development for the larger study, specific gaps in the literature were identified related to the issue of compassion training which fell outside the scope of the broader inquiry focused exclusively on defining and delineating the components of compassion. As a result, the research team modified the primary study interview guide during the protocol development stage (Table 1) to include questions focused on training. A further rationale for conducting a secondary analysis of this data subset from the sample population rather than conducting a separate study was to mitigate respondent burden by having palliative patients participate in multiple studies. As a result, we decided at our protocol development meeting to intentionally target a large qualitative sample to generate sufficient data to conduct this separate secondary analysis. While sample sizes are not pre-determined in qualitative studies, based on our methodological expertise and experience conducting robust qualitative studies we set an ambitious target of fifty patients, but in actuality fifty-three patients were needed to reach data saturation (Table 2).

**Table 1:** Guiding questions utilized in semi-structured interviews

1. What are the things that you have found to be important to your wellbeing during your illness? Particularly as it relates to the care you have received? 2. In terms of your own illness experience, what does compassion mean to you? 3 .Can you give me an example of when you experienced care that was compassionate? 4. How do you know when a healthcare professional is being compassionate? 5. Since you have had cancer, has compassionate care always been helpful? Have been there times when health providers’ efforts to be compassionate missed the mark? 6. What advice would you give health care providers on being compassionate? [Do you think we can train people to be compassionate? If so, how]? 7. We have talked about compassion, another word that might be related to compassion is sympathy. In your experience are compassion and sympathy related? [Tell me how they are the same or different]. 8. We have talked about compassion and sympathy, another word that might be related to compassion is empathy. In your experience are compassion and empathy related? [Tell me how they are the same or different]. 9. How does what you have told me about compassion relate to your experience of spirituality? 10. Is there anything that that we have not talked about today that we have missed or you were hoping to talk about?	

Grounded theory, an inductive qualitative research method, was used to collect data and analyze data concurrently through the three stages of Straussian grounded theory (open; axial and selective coding) [37, 38]. The rationale for conducting secondary analysis in grounded theory was espoused by Glaser over 50 years ago [39] and has been utilized by researchers across grounded theory traditions in the years following [40, 41]. The purpose of a secondary analysis is to analyze previously collected data from a primary study in order to explore a separate but related research question to either generate new theories or preliminary data for future grounded theory studies [39–41]. Patient participants were recruited via purposive sampling and theoretical sampling, whereby researchers intentionally sample certain individuals who are under-represented in the study sample or whose views are particularly important, thereby ensuring a heterogeneous sample and to develop a comprehensive understanding of the topic [37]. Patients were eligible to participate if they were: 1) at least 18 years of age; 2) able to speak and read English; 3) had a diagnosis of incurable cancer; 4) had no demonstrable signs of confusion (as determined by their clinicians); 5) and had a life expectancy of less than 6 months.

### Data collection and analysis

Data was collected from May to December 2013 from a hospital based palliative care consult team and a dedicated palliative care unit at a large urban academic hospital in Western Canada. Potential study participants were initially approached by a member of their palliative care team and informed of the study to gauge their initial interest. Interested participants were referred and then visited by a seasoned research nurse who provided additional information on the protocol, answered questions, and determined if patients were still interested and met entry criteria. Study participants then provided written informed consent, and a mutually agreeable time and place for an audio-recorded interview was determined. This study was approved by the Conjoint Health Research Ethics Board at the University of Calgary after a delegated review was conducted.

Audio-recorded semi-structured interviews were conducted by the research nurse in a private setting within the hospital and ranged between 45 to 90 min, with demographic data being collected after the interview (Table 2). An interview guide (Table 1) was developed based on the literature review [21], with additional questions and probes being interjected within the interview based on participants’ responses to the guiding questions. Non-verbal (eg. emotions) and contextual content (eg. environmental factors effecting the interview) of the interview were captured in the research nurses’ field notes. Grounded theory studies are driven by emerging data, with questions evolving over time as new concepts and ideas emerge. After interviewing 10 patients, the interview guide was modified to enhance clarity and expand the scope of inquiry based on patient feedback, being further revised at a face-to-face team meeting after interviewing 23 patients (Table 1). In relation to this study specifically, one of the questions we initially asked patients ‘Do you think we can teach people to be compassionate?’ was modified after 10 interviews as the terminology of ‘teaching’ seemed to conjure images of didactic learning which some patients felt was antithetical to the topic, preferring the language of cultivating compassion or compassion training (Table 1, question 6). Audio-recorded data were transcribed verbatim with content verified by a member of the research team who compared each line of the transcript with the corresponding audio file. All raw data was stored in a locked cabinet in the principal investigator’s office for a period of five years, then will be destroyed.

**Table 2:** Demographic information for 53 participants. Numbers expressed as percentages, unless otherwise stated

Mean age (Years)	61.44
Men	35.19
Women	64.81
Mean (Range) time between interview and death (Days)^a^	79.56 (8–261)
Marital status	
Never married	3.70
Married	59.26
Common law/Cohabiting	11.11
Divorced	16.67
Separated	0.00
Widowed	7.41
Other	1.85
Main social support^b^	
Spouse/Partner	66.67
Parent(s)	18.52
Sibling(s)	44.44
Children	70.37
Other relative(s)	14.81
Friend(s)	59.26
No One	3.70
Other	24.07
Religious and spiritual status	
Spiritual and religious	53.70
Spiritual but not religious	37.04
Religious but not spiritual	3.70
None	5.56
Highest education level attained	
No formal education	0.00
Elementary - Completed	1.85
Some high school	16.67
High school - Completed	9.26
Some university/College/Technical school	20.37
University/College/Technical school - completed	38.89
Post-graduate university - completed	12.96
Household net income	
≤$60 000/year	29.62
>$60 000/year	70.38

^a^Based on 45 patients that died at the time of analysis

^b^The total for these categories exceeds 100 % because patients were permitted to provide more than one response

Analysis of this data subset occurred through the three stages of Straussian grounded theory: open coding, axial coding and selective coding [38], in conjunction with analysis of data from the overall study. The analysis team, consisting of four investigators (SS, SR, TH, SM) with extensive qualitative expertise analyzed the data using the constant comparative technique [38]. The first stage of analysis, open coding, involved each team member independently reading transcripts line by line to discover, name, and organize phenomena through the generation of substantive codes by utilizing participants’ own words [37]. The second stage of analysis, axial coding, involved members of the research team coming together to rigorously code data, develop consensus on individual codes, compare data across interviews, and assign data to clusters or larger categories [37]. This was achieved by developing a coding schema that illustrated the context in which each category and theme occurred, the strategies in which it was managed, and the underlying conditions influencing the themes and categories [43]. The final stage of analysis known as selective coding, involved developing theoretical constructs from the data and identifying the relationship between categories, validating established relationships, and refining the categories as required [37]. This process generated a separate and unique pool of codes, themes, categories and exemplars related specifically to training which were subjected to a secondary inductive analysis by members of the research team (SS, MT, TH, SM, SR) to gain an in-depth understanding of the various concepts and their relationship to one another, which are reported for the first time here.

## Results

Three overarching categories, each containing three themes, emerged from the data: compassion aptitude, cultivating compassion and training methods (Table 3). Supporting verbatim quotes illustrating each category and theme were selected based on their representation of the views of the sample as a whole, while also honouring incidences where there were contrasting viewpoints.

**Table 3:** Overarching categories and themes derived from study data

1) Compassion aptitude: Intrapersonal factors impacting learners at baseline • The innate factor: A baseline for compassion • Vocational motivators and life experience: Inhibitors and facilitators of compassion • Embedded Resources: Eliciting and enhancing health care providers’ capacity for compassion 2) Cultivating compassion: Recommended essential skills for developing compassionate care providers • Building a relationship • Understanding the patient as a human being: Seeing the person behind the disease • Emotional resonance: Developing a human connection 3) Training methods • Person-centered communication skills • Reflective practice • Compassionate role modeling	

### Compassion aptitude: intrapersonal factors impacting learners at baseline

The majority of participants felt that while training healthcare providers in compassion was possible, learning was contingent on two factors: 1) the attributes and motivators that learners brought with them at baseline; and 2) the nature of the topic in general, requiring an experiential learning approach focused on the cultivation of compassion in contrast to a more traditional teaching or competency based approach.

#### The innate factor: A baseline for compassion

The majority of participants felt that a learner’s capacity for compassion was partially dependent on the innate attributes that healthcare providers possessed prior to embarking on their healthcare training. Some participants understood the innate nature of compassion in an ‘either or’ fashion, but most participants felt that while learners’ aptitudes varied at baseline, it could nonetheless be nurtured to varying degrees. In describing this, participants often spoke of compassion as originating from the heart or the character of the learner - in contrast to their intellectual abilities or professional duties - involving both ‘doing for’ and ‘feeling for’ the patient (Table 4).

**Table 4:** Category 1 - Compassion aptitude: Intrapersonal factors impacting learners at baseline

Theme 1: The innate factor: A baseline for compassion “Some people have it naturally, some people don’t” (Participant 10) “They have to have heart and not lose it. They have to have the heart to ‘be with’, not everybody does” (Participant 25). “It’s hard because some people just aren’t compassionate, they don’t give a crap about anybody. I mean I know there are people out there that are, and there are miserable people out there” (Participant 23). Theme 2: Vocational motivators and life experiences: Inhibitors and facilitators of compassion “The patients need you for more than just what you can do medically for them. I think you need to be there because you love what you do, whatever that is, you just you have to love what you do. You have to love those patients, be compassionate…Don’t come in for the money…It’s something they’re grown up with, they’ve learned over the course of their lifetime and this sort of thing. I think possibly that’s one of the reasons why they’ve gotten into the health care industry.” (Participant 21) “I think in order to be compassionate, there's a certain value in having a background experience in your family life, as you grew up, a variety of experiences that enable you to understand people” (Participant 44). “They should want to be in that place because they want to be in that place, they want to be helping, they want to be working with people in the situation that they’re in, not because they’ve been assigned… I think for some people they just, if they never had any compassion in their lives, they’ll have a hard time” (Participant 10). Theme 3: Embedded resources: Eliciting and enhancing healthcare providers’ capacity for compassion “It’s how you live your life…you’re born with a bit of it but it doesn’t come, you have to work at it as well” (Participant 38). “You can plant the seeds and through experience, it can grow” (Participant 8). “I think we're gifted with different amounts of intuitiveness, and we are gifted in a sense of some people find it more easier more than others do… I think we have to teach them there are techniques.” (Participant 47). “It’s there, the compassion is there already, it just needs to bloom” (Participant 49).	

#### Vocational motivators and life experience: Inhibitors and facilitators of compassion

While patients acknowledged that learners’ capacity for compassion was contingent on pre-existing innate qualities, they identified vocational motivators and life experiences as additional influencers that could either foster or diminish these underlying qualities. Participants felt that personal experiences of compassion or the lack thereof, whether as a recipient, a giver, or as a mentee were particularly powerful teaching moments that informed and impacted healthcare provider’s ability to provide compassionate care. Conversely, participants felt that inhibiting vocational motivators such as finances and career advancement could potentially usurp the virtue-based motivators that patients felt were the impetus for originally pursuing a healthcare career (Table 4).

#### Embedded resources: Eliciting and enhancing healthcare providers’ capacity for compassion

Participants felt that compassionate care was influenced by pre-existing qualities, life experiences, and vocational motivators. Participants felt that within a supportive teaching environment, these embedded qualities could be nurtured over time (Table 4). They endorsed experiential modes of learning over didactic approaches for healthcare providers engaged in honing their capacity and skills in providing compassionate care.

### Cultivating compassion: Recommended essential skills for developing compassionate healthcare providers

Study participants described a number of core competencies they felt were essential to compassion training and practice: building a relationship, understanding the patient as a human being, and developing a human connection.

#### Building a relationship

Patients identified healthcare providers’ ability to build a relationship with their patients as a core component of compassion-based training. Building a relationship with patients involved healthcare providers’ ability to develop trust, dialogue, and show genuine interest in the person in their care, versus a strictly objective approach that restricted the relationship to clinical matters. While patients did not feel that establishing a relationship was an antecedent to compassionate care, they did feel that compassion was fostered and optimized through relationship. As a result, patients stressed the necessity for healthcare providers to receive training in the development of interpersonal skills in order for compassion to flourish (Table 5).

**Table 5:** Category 2 – Cultivating compassion: Recommended essential skills for developing compassionate care providers

Theme 1: Building a relationship “Look at (your patient) as someone you want to build a relationship with. If you don't know the answer, don't pretend to know the answer. Because the minute you pretend to know the answer, there goes the relationship right out the window” (Participant 40). “Try to create as much as possible for the time that they’re in here, a relationship” (Participant 51). Theme 2: Understanding the patient as a human being: Seeing the person behind the disease “Appreciate all of the different facets and parts of a person’s life that are affected, not just the illness. Let’s look at the whole picture. Their love life, their family life, their supports, their job, and the huge financial loss” (Participant 19). “You’re dealing with human beings and everything else, not case files and or anything else, that these are actual human beings with families and children. Try to understand that and put aside what you know in medical school, what you've learned about these diseases and everything else and not treat people as a case files they’re actually human beings” (Participant 43). “See each person as an individual and make it known to that person that they see them as an individual” (Participant 48). “I just think you have to take time to realize each individual patient is different. Each person is an individual and we all should be treated individually. Just take a breath and realize that they're human and they're sick. I think the health care providers need to realize that you're here for a reason” (Participant 45). Theme 3: Emotional resonance: Developing a human connection “They have to learn how to be in tune with people in the moment” (Participant 48). “Before your actions, before you speak, before you do something, I would rerun it in your head. Put yourself in that person’s position” (Participant 50). “Feel the feeling of the sick person, just be familiar with whom you are serving” (Participant 49). “Part of your job as a doctor, is to sit down with people when you’re giving them whatever diagnosis it might be, to ask them if they have any questions, to ask them if there’s anybody that they can call, ask what can we do for you now, where are we going to go now, and if you don’t have the answer as a doctor, you have your colleagues of doctors that you can go to” (Participant 5). “Nurses are dealing with people, they should be able to read people and understand people and talk to people and of course, the worst thing of all is dealing with really aggravating people, to even get over that hump and try to help where they can” (Participant 9).	

#### Understanding the patient as a person: Seeing the person behind the disease

Most participants felt that approaching and understanding the patient as a fellow human being was a critical aspect of compassionate care. Many participants felt that the emphasis in healthcare education on the biomedical model inadvertently caused students to view the patient as a body or disease, rather than a person. In identifying the importance of a person-centred approach, many patients contrasted this with experiences where this was lacking, sharing clinical encounters where they felt they were treated as an object, a number, or a disease. While participants recognized that developing biomedical expertise was a learning priority, they also indicated that greater effort needed to be made in educating healthcare providers about acknowledging and addressing their holistic needs (financial, social, emotional, spiritual). Compassionate care, according to most patients, involved addressing their medical needs within the larger backdrop of the person, including the systemic effect that their illness had on other domains of their life (Table 5).

#### Emotional resonance: Developing a human connection

While healthcare providers’ ability to build a relationship and understand the patient as a person were foundational skills, participants felt that healthcare providers needed to cultivate emotional resonance —the ability of healthcare providers to ‘feel for’ their patients, in order for a relationship to be considered compassionate. In describing this core competency, patients shared exemplars of compassionate care involving nurses, physicians and allied health professionals who were particularly skilled at ‘tuning in’, ‘reading people’, and ‘feeling for’ their suffering person. Emotional resonance not only involved healthcare providers’ ability to relate and position oneself ‘in the patients shoes’, it also required healthcare providers to actively seek to understand the patient in an ongoing and proactive manner. Participants described a number of physical gestures that implied emotional resonance, including proximity to the patient (sitting vs. standing), making eye contact, and physical touch. While patients felt these techniques could be taught, they cautioned against a prescriptive approach, that while mirroring compassionate behaviours, could be experienced as disingenuous (Table 5).

### Training methods

Participants suggested three primary teaching methods to effectively train healthcare providers in the provision of compassionate care: person-centred communication skills; reflective practice; and role modeling. There was some variance among individual participants related to the three methods that emerged from the data, with some patients suggesting all three methods while others focused on one or two. Participants were unequivocal in their belief that didactic, textbook, or traditional competency-based approaches were not conducive to compassion training. According to patients, the personal and relational nature of the topic required a more experiential, heuristic, and learner-centred approach.

#### Person-centered communication skills

Participants identified person-centered communication as a foundational skill that healthcare providers needed to develop in order to provide compassionate care. In addition to traditional notions of person-centred communication that is respectful and responsive to patient preferences, needs and values, patients identified a number of additional features specific to compassion. Although participants did not identify a particular method of learning, participants felt that clinical communication training should focus on the development of interpersonal skills that fostered a relationship that extended beyond simply the sharing of clinical information. In addition to training healthcare providers in actively listening and seeking to understand the broader meaning that clinical information had on patients’ lives, compassionate person-centred communication required healthcare providers to not simply engage the patient as a person but to see themselves as a person within the clinical encounter. Compassionate communication was often described by patients as being conveyed through healthcare provider virtues, such as sincerity, love, openness and honesty, which required healthcare provider training on how to effectively cultivate and appropriately incorporate their virtues into their care (Table 6).

**Table 6:** Category 3 – Training methods

Theme 1: Person-centered communication skills “Communication is the number one ingredient in every way shape and form in their connection with the patient” (Participant 34). “Don’t be condescending. I mean you can be calming without kind of feeling like you’re talking down to them. Just speak to them normal…we tend to do that to people who are sick, we start whispering, we start treating them different and it’s the worst thing. Just be open, because in my experience, that’s what people want…. listen to what’s underneath what they’re saying .” (Participant 23) “Be prepared to listen to what they have to say” (Participant 36). Theme 2: Reflective practice “To really think first and really stop and think about the situation, if this was me or if this was my mom or if this were that, or maybe go back to a time where I had a moment that was uncomfortable to me and what I felt like, to really before you just go there” (Participant 27). “We learn from experience, and so if we can somehow get them in an awkward situation, where that person is not hearing what they’re saying and is going off on different things and then the vice versa, Having students sit in a difficult situation and having someone listen and stop and comprehend and help them address, I think those things are very teachable.” (Participant 10) “We’re changeable, and you know, first you have to know where people come from” (Participant 8). Theme 3: Compassionate role modeling “It’s not just the communication courses, people also need to see compassion demonstrated” (Participant 48). “Role playing would be one way which you could do that, you would have your exemplar, and have them mimic, copy those particular kinds of behaviors” (Participant 51). “Showing people that here's how somebody's reacting to a person or something and show them that this is you know, you just made the patient feel like he's a burden to you now. That the patient isn't there because he wants to be, he’s there because he's sick and he needs your help. And you're there to give him that help, that’s why you're there.” (Participant 33) *“*I think you can do your best to you know show examples of what compassion could look like” (Participant 25).	

#### Reflective practice

Participants identified the need for learners to reflect on their personal and professional experiences as an important teaching method. In addition to critically reflecting on their clinical practice, participants identified the need for healthcare providers to develop a practice of self-reflection as an essential competency of compassionate care. Participants suggested that self-reflective exercises that invited learners to position themselves in the patient’s situation and to focus on their personal beliefs and values related to suffering, death and dying as particularly salient teaching methods. Participants also identified journaling, viewing media centered on compassion, reviewing case studies, informal conversations with patients, and reflective rounds with their fellow students as additional methods (Table 6).

#### Compassionate role modeling

Participants frequently identified shadowing or learning from compassionate role models as another potential teaching method. Patients reflected a belief that not only was compassion conveyed through role modeling, but so too were non-virtuous bedside attributes such as apathy, disrespect and contempt. Participants felt that compassionate role models allowed trainees to both emulate exemplary practices, and to critically examine areas where their compassionate clinical practice may be underdeveloped. Participants felt that role modeling could potentially have an exponential and transformative effect in clinical practice, with trainees subsequently role modeling compassion to other healthcare providers. Participants also identified role-play as another method for developing the skills associated with compassion. In addition to allowing learners to practice their clinical skills, participants felt that having them play the role of the patient was particularly important in compassion training (Table 6).

## Discussion

This study is the first study of patient perspectives on training healthcare providers in compassion. While participants in this study were not pedagogical experts, their life experience, their proximity to suffering, death and dying, and role as the recipients of compassion provide an invaluable perspective to aid in the development of compassion training. In addition to insight on the importance of compassion training in palliative care, participants emphasized the importance of compassion across the disease trajectory, imploring all health care providers to demonstrate practice competency in this area.

Results suggest that while elements of compassion can be taught, enhancing learner’s capacity for compassion is contingent on the aptitude that healthcare providers possess at baseline. These findings affirm the results of other studies which identified personal illness experiences among physicians [24], the inherent qualities of healthcare professionals and students [44–46], and the moral virtues of health care providers [22, 42, 47], as significant mediators of compassionate care. While patients felt that compassion aptitude varied across individual healthcare providers, they felt that learners’ capacity for compassion could nonetheless be cultivated, primarily through experiential learning methods. The importance and feasibility of developing compassion by nurturing the inherent qualities of learners affirms the findings of studies within the neuroscience that have revealed that compassionate feelings are activated and enhanced through contemplative practices [34–36]. In addition to affirming contemplative practice learning techniques, the current study, in identifying that compassion is predicated in action and associated skills and behaviours, emphasizes the necessity of an applied component aimed at effectively alleviating a person’s suffering, as without this the construct validity of such interventions and their clinical relevance is limited.

Reflective practice was identified as a potentially efficacious means for compassion training, building on reflective practice theory within the healthcare literature. The notion of reflective practice was coined by Schön as a clinical teaching method of revisiting a clinical experience for the purpose of debriefing and adaptive learning [48]. Reflective practice has traditionally focused on the enhancement of clinical skills, knowledge and attitude based on retrospective reflection rather than ‘reflection in action’ [49] A systematic review within healthcare education, concluded that it is an inherently difficult concept to measure with no consensus related to its efficacy in increasing self-understanding, changing clinical behaviours or enhancing learner competence [49, 50]. Participants in this study emphasized a two-pronged approach to reflective practice involving both reflecting on clinical practice and intrapersonal feelings in an ongoing manner, as a means of nurturing compassion in healthcare. These results are consistent with recent studies of healthcare students and practicing clinicians, which report that reflecting on personal beliefs, personal experiences with illness, and experiences of receiving compassion, act as catalysts for developing compassion [23–26, 51, 52].

Participants in this study also identified the integral role that clinical role models play in compassion training, echoing the findings of a qualitative study of medical students [25]. In contrast to these previous studies, participants in our study felt that role models were not just a powerful conduit for developing compassion, but could equally function as a barrier to compassion, eroding learners’ virtuous qualities over time [53]. Burack et al. reported similar findings in a study investigating the role of attending physicians in compassion training, discovering that attending physicians were reticent to address clearly identifiable non-compassionate behaviours among their students [54]. Finally, participants in our study and studies exploring compassion training from the perspective of nursing instructors, emphasized that developing compassion in learners was not conducive to a competency-based approach, suggesting that challenges related to compassion training may have less to do with feasibility and more to do with the content and teaching methods employed [44, 55].

Results suggest that compassion training may require a reconceptualization of person-centred care, which in addition to healthcare providers seeing the patient as a person, requires them to reflect on how their own personhood impacts the clinical relationship—person-to-person communication [56]. Compassionate communication not only involved communicating clinical information in a timely and accurate manner that was sensitive to the patients beliefs and values, but having healthcare providers share aspects of themselves, while also actively placing themselves in their patient’s shoes. This is consistent with other studies, which identify relational skills such as getting to know the patient, emotional resonance, and conveying a genuine sense of care toward the patients as foundational markers of compassion [23, 26, 47, 51, 57–59].

Developing an evidence-informed understanding of compassion and subsequent educational interventions has significance for the training of current and future healthcare providers [5, 6, 19]. Our findings suggest that interventions need to include clinical components, in addition to affective training focused on the cultivation of compassionate feelings--as compassion is manifested in application that extends beyond the self. Since compassion is individually expressed and experienced, a learner centred approach to training that incorporates direct patient feedback is imperative to insure learning objectives are met and have their intended effect on recipients. Experiential learning interventions aimed at the development of a reflective practice, both personal and professional, seem to be an essential skill that are conducive to a classroom setting. However, since the practice setting is a poignant mediator of the sustainment of these skills overtime, interventions need to especially target the role of clinical preceptors and mentors.

By way of clinical implications, since compassion and adverse states of apathy, disregard and ambivalence seem to be learned in the practice settings, practicing healthcare providers and administrators are implored to ask themselves whether their practice and the clinical culture causes trainees compassion to flourish or falter. The current findings emphasize the need for future research and theory development in this area while also underscoring the importance of addressing these detrimental gaps in health care education [19]. There is a need for further research focused on developing clinical compassion measures to evaluate the effect of compassion training in clinical practice and the retention of training over time. Subsequent educational research is needed to pilot, develop, and validate compassion-training interventions in order to evaluate their clinical efficacy. There is also a need to replicate this study in other healthcare populations and care settings, in order to tailor education interventions to learners, their practice settings and the diverse patient population they serve. Finally, in terms of limitations, as this is a qualitative study of Canadian advanced cancer patients, the generalizability of this study is limited as experiences and understandings of patients in other locations and within other disease groups likely vary. Second, while the study population is representative of the patient population within this setting, they were highly educated (72 % at least some University) which may have inadvertently skewed the importance and need for a formal educational approach to compassionate training. This may have been further influenced by our study setting-- a large teaching hospital were patients received care from trainees which may have introduced a response bias. Finally, while this was a robust study in terms of sample size and methodology, it is nonetheless a secondary analysis that requires further study dedicated to the topic of compassion training exclusively, including the development of a theoretical model in order to test future educational interventions.

## Conclusions

Compassion is fundamental to the delivery of quality healthcare. This novel study highlighted the importance and significance of compassionate care from the perspectives and experiences of advanced cancer patients, identifying salient teaching methods deemed important in the provision of compassionate care. The results drawn from this study inform future inquiries focused on knowledge transfer in devising curricula and tools to train and educate current and future health care providers in compassionate care. Understanding how compassionate care can be taught and optimally provided may better strengthen patient-provider relationships, enhance therapeutic interventions, and ultimately improve patient care.
